# HOME IS WHERE THE HEART IS: OPTIMIZING AND TAILORING HOME AND COMMUNITY-BASED SUPPORT FOR DEMENTIA CAREGIVERS

**DOI:** 10.1093/geroni/igz038.2026

**Published:** 2019-11-08

**Authors:** Quincy M Samus, Nancy Hodgson, Joseph E Gaugler

**Affiliations:** 1 The Johns Hopkins University, Baltimore, Maryland, United States; 2 University of Pennsylvania, Philadelphia, Pennsylvania, United States; 3 University of Minnesota - School of Public Health, Division of Health Policy and Management, Minneapolis, Minnesota, United States

## Abstract

Family caregivers, often “de facto” members of the care team for persons with dementia, play a central role in ensuring safety, support, quality of life, and continuity of care. Most often, they provide this care for loved ones at home and over a long period of time, as the illness progresses and care need intensifies. This session will provide a unique understanding of potential ways to optimize support for family caregivers in provision of day-to-day care in the home by examining often-overlooked factors influential in the health and wellbeing for both caregiver and persons with dementia. Presentations will draw from three large community-based trials testing interventions to support dementia caregivers. Drs. Samus and Sloan will present on common unmet needs identified by family caregivers and explore how needs differ by disease stage and race. Dr. Fortinsky will present baseline caregiver care-related challenges in a diverse cohort of caregivers and the effects of a caregiver intervention designed to mitigate these challenges. Dr. Hodgson will present the common symptoms clusters among home-dwelling persons with dementia patients and how these are associated with caregiver distress. Dr. Jutkowitz will discuss factors influencing dementia caregiver’s willingness to pay for help at home. Findings from this session will help elucidate care needs that matter most to family caregivers in diverse community-living cohorts and how we might optimize and tailor supportive home-based interventions to target these needs and challenges.

